# P-2200. Public Health Partnerships for Hepatitis C Elimination: Using Telemedicine and Syringe Service Program Hubs to Overcome Treatment Barriers in Rural Kentucky

**DOI:** 10.1093/ofid/ofae631.2354

**Published:** 2025-01-29

**Authors:** Nicholas Van Sickels, Jaime Soria, Amanda B Wilburn, Jana Collins, Kendal Welty

**Affiliations:** The University of Kentucky, Lexington, Kentucky; University of Kentucky, Lexington, Kentucky; The University of Kentucky, Lexington, Kentucky; University of Kentucky, Lexington, Kentucky; University of Kentucky, Lexington, Kentucky

## Abstract

**Background:**

People who inject drugs (PWID) in rural areas of the United States face multiple barriers accessing Hepatitis C (HCV) care, including insurance-based treatment restrictions, long travel distances, and housing instability. The Kentucky Income Reinvestment Program (KIRP) provides harm reduction services, HIV and HCV testing, and linkage-to-care across most of Kentucky's Syringe Services Programs (SSPs). KIRP, in partnership with three high-prevalence counties (Figure 1) sought to reduce HCV treatment barriers by using SSPs as a hub for telehealth appointments, allowing for delivery of medications to the SSP, and providing inter-visit support to participants. We present early outcomes from this model and examine its effects on treatment initiation.

**Figure:**
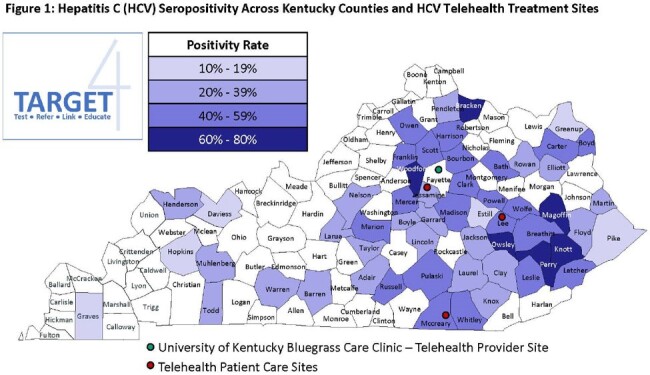

**Methods:**

Advertising for the program began in McCreary County in June 2023, with Jessamine and Lee counties starting in January 2024. Patients were scheduled (or could walk-in) one weekday afternoon at each SSP. Visits were conducted via audio-visual (AV) telehealth, with KIRP staff present for support. HCV evaluation followed guideline-endorsed simplified pathways for those meeting criteria. Labs were performed at a 3^rd^ party lab, and patients were followed using a minimal monitoring protocol.

**Figure:**
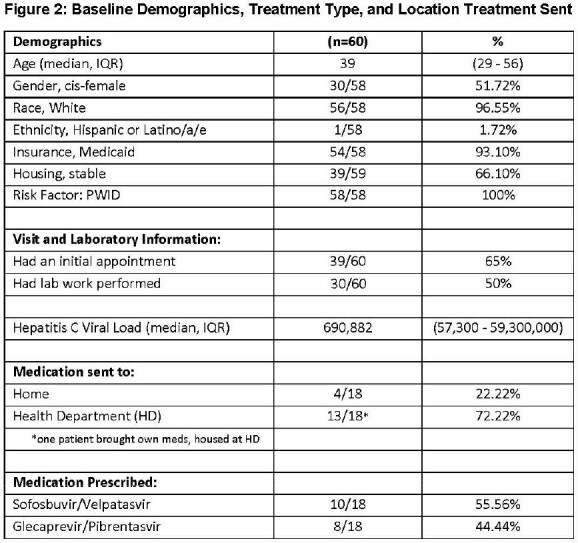

**Results:**

Between June 1, 2023 and April 30, 2024, 60 HCV seropositive patients were assessed, with 39 seen via AV telehealth. The median age was 39; 96% were White, 51% identified as cis-female, 93% were Medicaid recipients, and 34% reported unstable housing (Figure 2). Of 39 patients seen, 30 had labwork completed, 22 were RNA positive, and 18 initiated treatment. To date, ten patients completed treatment and one achieved SVR (Figure 3). Patients preferred to receive or house medications at the health department (78%). No significant effects of baseline demographics were found on the outcome of treatment initiation.

**Figure:**
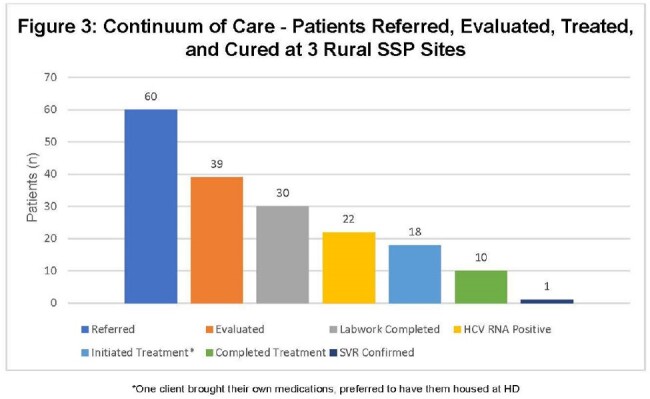

**Conclusion:**

At baseline, less than a third of Kentuckians achieve a cure for HCV. Early findings from our intervention in three high-prevalence counties suggests the use of telehealth and an SSP hub can increase HCV treatment initiation and possibly completion for those who attend a visit. Further study of streamlined care delivery is needed to overcome HCV treatment barriers, especially in areas with limited healthcare access.

**Disclosures:**

All Authors: No reported disclosures

